# Gamma Rhythms in the Brain

**DOI:** 10.1371/journal.pbio.1001045

**Published:** 2011-04-12

**Authors:** Xiaoxuan Jia, Adam Kohn

**Affiliations:** 1 Dominick P. Purpura Department of Neuroscience, Albert Einstein College of Medicine, Bronx, New York, United States of America; 2 Department of Ophthalmology and Visual Sciences, Albert Einstein College of Medicine, Bronx, New York, United States of America

Brain rhythms are activity fluctuations shared in populations of neurons. They are evident in extracellular electric fields and detectable through recordings performed within the brain or on the scalp. The gamma rhythm, a relatively high frequency (30–80 Hz) component of these fluctuations, has received a great deal of attention. Gamma is modulated by sensory input and internal processes such as working memory and attention. Numerous theories have proposed that gamma contributes directly to brain function, but others argue that gamma is better viewed as a simple byproduct of network activity. Here we provide a basic introduction to this enigmatic signal, the mechanisms that generate it, and an accompanying paper in *PLoS Biology* attempting to elucidate its potential function.

Hans Berger first successfully measured the brain waves of humans in 1924 using the electroencephalogram (EEG) [1]. His goal was to demonstrate that the electromagnetic fields of the human brain could be used for telepathy. Although the signals he detected were unsuccessful for this purpose, the EEG was widely adopted by clinicians and scientists. This is because the recordings are easy to perform and the rhythms detected are informative of brain state. For example, when we are in a deep sleep, the EEG consists of low-frequency, large-amplitude oscillations; when we are awake and attentive, it consists primarily of fast, small amplitude rhythms.

Brain rhythms are evident as extracellular voltage fluctuations. These arise from summed electrical activity (primarily, but not exclusively, inputs) in populations of neurons, and are shaped by the geometry and alignment of those neurons [2]. The resultant fluctuations can be measured on the scalp by EEG or magnetoencephalography (MEG), and intracranially with subdural electrodes (electrocorticography). They can also be measured, on a more local basis, with a high impedance electrode placed in the brain (Figure 1A). The voltage fluctuations detected are then low-pass filtered (<250 Hz) to capture the slower fluctuations of brain rhythms (Figure 1B). The resultant signal—termed the local field potential (LFP)—was frequently used to study brain function, until it fell in popularity with the advent of single-cell electrophysiology in the late 1950s. Over the last decade, however, LFPs have attracted renewed interest as a potentially useful signal for studying the behavior of ensembles of neurons.

**Figure 1:**
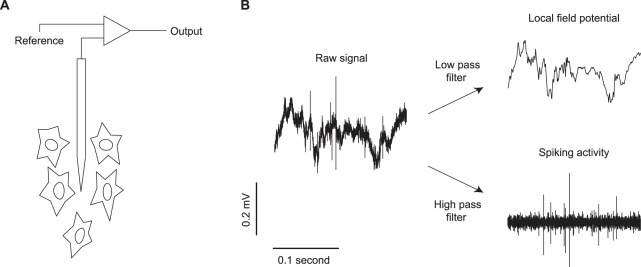
Illustration of LFP recordings. (A) A high impedance electrode detects extracellular electrical activity of nearby neurons. (B) This raw signal is low-pass filtered (e.g., <250 Hz) to provide the local field potential (LFP), and high-pass filtered (e.g., 0.5–10 kHz) to isolate spiking activity.

The LFP is a continuous voltage signal that can vary in amplitude and frequency content. Like the EEG, it can be decomposed into different frequency components—delta (<4 Hz), theta (4–8 Hz), alpha (8–12 Hz), beta (12–30 Hz), gamma (30–80 Hz), and high-gamma or high-frequency activity (>80 Hz)—although the precise frequency ranges associated with these terms vary across studies. The relative contribution of these different components to the measured signal is quantified by their relative power (Figure 2). In quiescent networks, most of the power in the LFP is found at low frequencies, indicating that rhythms like delta and theta contribute more significantly than high frequency ones. This is still the case when networks are activated, but less so: the power in higher frequencies increases, whereas that in lower frequencies is suppressed. The enhancement of gamma power in this driven state is particularly striking and is evident as a distinct “bump” in the power spectrum (Figure 2; right panel, solid line).

**Figure 2:**
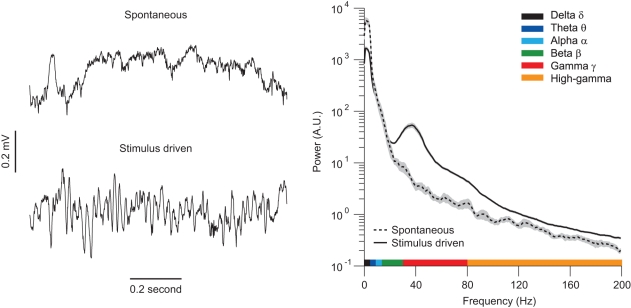
LFPs for spontaneous and stimulus-driven activity. (Left) Example traces of the LFP during spontaneous activity and visually driven activity in primary visual cortex. (Right) The corresponding power spectra for the two conditions, with the frequency ranges of different rhythms indicated.

A prominent gamma rhythm provides a signature of engaged networks. Gamma has been observed in a number of cortical areas, as well as subcortical structures, in numerous species. In sensory cortex, gamma power increases with sensory drive [3],[4], and with a broad range of cognitive phenomena, including perceptual grouping [5] and attention [6]. At a given recording site, gamma is stronger for some stimuli than others, generally displaying selectivity and a preference similar to that of nearby neuronal spiking activity [7],[8]. In higher cortex, gamma power is elevated during working memory [9] and learning [10]. Interestingly, irregular gamma activity has been observed in neurological disorders such as Alzheimer's disease, Parkinson's disease, schizophrenia, and epilepsy [11].

To interpret the meaning of changes in gamma requires an understanding of the cellular and network mechanisms that generate it. Fast-spiking GABAergic inhibitory interneurons are known to be crucial, with their activity being both necessary and sufficient to generate gamma [12]–[14]. Network models suggest that this process may be enhanced by interactions with excitatory neurons [15] and that local gamma-generating networks can be coupled by long-range horizontal connections [16] or gap junctions among inhibitory interneurons [17]. Such coupling would seem necessary, as gamma has been shown to be coherent across millimeters of cortex [18]–[20].

It is well established that gamma correlates with engaged or driven networks, but it is less clear whether it is a simple byproduct of network activity or has an important functional role. This is not for lack of proposals: numerous functions have been attributed to this rhythm. Most of these hinge on a relationship between gamma and the timing of spiking activity in nearby neurons. Spikes are actively generated signals in individual neurons and relay information between neural networks. Gamma activity is not actively propagated. It is a component of an extracellular field potential that reflects primarily the synaptic input to a collection of neurons. Because of this, gamma can only play a role in processing if it is linked to spiking activity in a meaningful way. A coupling between gamma and spike timing could arise because local inhibitory neurons—which contribute strongly to gamma—fire preferentially at the trough of the gamma cycle [21]. This makes the spiking of excitatory projection neurons more likely to occur at an offset phase, when inhibition is weaker.

Based on this mechanism—in some cases, predating its discovery—numerous theories have suggested that the gamma coordination of spiking activity is central to cortical processing. One purports that gamma acts as a temporal reference frame, with the gamma phase at which spikes occur encoding stimulus strength [22]. Consistent with this suggestion, neurons in visual cortex can encode stimulus orientation in “phase-of-firing” relative to gamma [23]. Another theory proposes that gamma may influence the communication between neuronal populations [24],[25]. Here the suggestion is that when field potentials and spiking activity in two groups of neurons are phase coherent, the communication between them will be maximal. A third hypothesis, “binding by synchrony”, suggests gamma can link the representation of a single sensory input (e.g., a visual object) whose features are processed by different groups of neurons [26]. At the heart of these proposals is the concept that gamma influences spike timing and that this affects cortical computation and function.

A number of recent studies have taken a more critical view of the role of gamma, testing whether it has the properties required for its purported functions. One study showed that the frequency of gamma can vary between nearby sets of neurons, limiting its ability to function as a global timing reference [27]. Another has shown that, at a single site, the gamma rhythm is not “auto-coherent”, meaning that its absolute phase changes with time, a pernicious feature for a reference clock or integrative signal [28]. In this vein, it is also worth noting that gamma fluctuations are small, roughly 10–20 microvolts on average, and account for only 0.5%–10% of the total power in the LFP. These observations raise the possibility that gamma is simply a resonant frequency that has no special function, a byproduct of a recurrently connected neuronal network.

To test existing proposals, and to understand the function of gamma more generally, it is critical to analyze the temporal relationship between spikes and gamma. This is typically done using spike-field coherence analysis or by spike-triggered averaging of the LFP, both of which provide a measure of the temporal or phase relationship between spike trains and the LFP. These measures have revealed weak but measurable coupling, which increases when gamma power is elevated [6],[9],[29]. This coupling is only meaningful, however, if the two signals are measured independently. LFPs and spikes are often recorded from a single electrode. Because extracellular action potential waveforms have a broad frequency spectrum, including power below 250 Hz, their energy can leak into the LFP signal [30],[31]. That is, the low-pass filtering of the extracellular voltage signal, which is used to isolate the LFP (Figure 1), may not entirely remove action potential waveforms. The resultant contamination would introduce spurious correlations: the timing of spikes will appear to be related to fluctuations in LFP power for the simple reason that a remnant of the spike waveform remains in that signal.

The paper by Ray and Maunsell in this issue of *PLoS Biology* carefully examines the interaction between spikes and the LFP [32]. Using clever analysis, they provide rigorous quantification of the contamination of LFP signals by spike waveforms and spike-related transients. They show this contamination can contribute to the high frequency components of the LFP, and has a measurable effect on frequencies extending down to 50 Hz. For studies that have focused on spike–gamma interactions in the lower gamma rhythm range (30–50 Hz), contamination is thus less likely to be an issue, although the precise frequency range over which contamination occurs will depend on the specific properties of the filters used to separate spikes from LFPs. However, for frequencies above this range (>50 Hz), which include the higher frequencies of gamma and the full range of high-gamma, spike–LFP correlations might be inflated by spike contamination. More generally, the findings of Ray and Maunsell suggest the need for a re-evaluation of the spike–LFP timing relationship, particularly in cases where this has been established using signals recorded from the same electrode.

In a related analysis, Ray and Maunsell tested the relationship between high-gamma and gamma power. To do so, they manipulated stimulus size. It is well known that many neurons in primary visual cortex are less responsive to large stimuli than small ones [33]. Gamma power, in contrast, increases with stimulus size [34]. Building on this work, Ray and Maunsell show that high-gamma power is modulated similarly to spiking activity by stimulus size (i.e., suppressed by large stimuli), and thus differently from gamma. The authors also show that high-gamma power has similar temporal dynamics as spiking activity, whereas gamma does not. Together, this strongly suggests that the proposed functions of gamma do not apply to high-gamma, even in situations where both signals are similarly enhanced. Rather, high-gamma may best be viewed as a reliable and convenient signal to represent multi-unit activity (MUA).

The findings of Ray and Maunsell help clarify the relationships among gamma, high-gamma, and spiking activity. But much remains unclear. It is certain that gamma, like other brain rhythms, can provide a signature of cognitive state, as well as network dysfunction. To move beyond the interesting correlation between these rhythms and brain state, like those first described by Berger, we need a better understanding of the underlying generative mechanisms, the way in which these signals modulate spiking activity, and the effect they have on the computations performed by neuronal networks. Only then will we know what role, if any, gamma plays in cortical function.

## Abbreviations

EEG: electroencephalogram
LFP: local field potential
